# Survival and infectivity of *Paragonimus westermani* Metacercariae in soy sauce–marinated crayfish

**DOI:** 10.1016/j.fawpar.2025.e00277

**Published:** 2025-07-18

**Authors:** Eun-Min Kim, Yan Jin, Sung-Tae Hong

**Affiliations:** a Department of Microbiology, Lee Gil Ya Cancer and Diabetes Institute, Gachon University College of Medicine, Incheon 21999, Republic of Korea; b Department of Microbiology, Dongguk University College of Medicine, Gyeongju 38066, Republic of Korea; c Department of Tropical Medicine and Parasitology and Institute of Endemic Disease, Seoul National University College of Medicine, Seoul 03080, Republic of Korea

**Keywords:** *Paragonimus westermani*, Soy sauce, Crayfish, Metacercariae, Paragonimiasis

## Abstract

*Paragonimus westermani*, a zoonotic lung fluke, causes respiratory symptoms resembling tuberculosis. In Asia, human infections typically occur through the consumption of raw or marinated freshwater crabs or crayfish containing *P. westermani* metacercariae. With increasing global food trade and occasional exposure during international travel, cases have been reported in non-endemic regions, raising significant food safety concerns. In this study, naturally infected freshwater crayfish (*Cambaroides similis*) were collected from Haenam, South Korea. Crayfish were marinated in soy sauce containing either 10 % or 20 % sodium chloride (NaCl) and stored at 4 °C for 1–60 days. *P. westermani* metacercariae were recovered at each time point and morphologically classified as viable, borderline (uncertain viability), or dead. To assess infectivity, 100 *P. westermani* metacercariae from each group were orally inoculated into beagle dogs (*n* = 3 per group), and worm recovery was assessed after 12 weeks. After 14 days of marination, the survival rates of *P. westermani* metacercariae were 83.3 % in 10 % NaCl and 2.0 % in 20 % NaCl soy sauce. Complete inactivation occurred after 60 days in 10 % NaCl and 30 days in 20 % NaCl. Infectivity tests showed worm recovery rates of 82.5 % (viable), 33.7 % (borderline), and 0 % (dead). These findings indicate that even high-salt, cold-storage marination does not guarantee parasite inactivation. Viable *P. westermani* metacercariae can persist in marinated crayfish under commonly used culinary conditions. These results underscore the risk of foodborne lung fluke infections and emphasize the need for clearer public health guidance regarding the consumption of undercooked or inadequately processed freshwater crustaceans.

## Highlights

• *Paragonimus westermani* metacercariae survive in soy sauce–marinated crayfish.
• High-salt marination (up to 20 % NaCl) does not ensure parasite inactivation.
• Infectivity persists even after partial morphological degradation.
• Borderline metacercariae can infect all animals, with ∼30 % worm recovery.
• Traditional marination methods pose a risk of foodborne paragonimiasis.

## Graphical abstract

**Figure:**
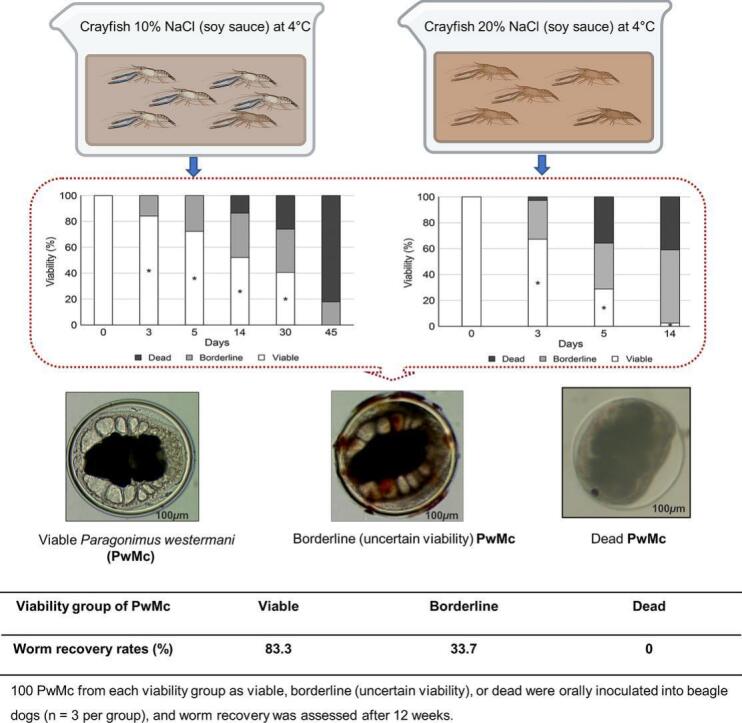

## 1 Introduction

*Paragonimus westermani*, is a zoonotic lung fluke that causes paragonimiasis, a parasitic disease often mistaken for pulmonary tuberculosis due to similarity in clinical symptoms and radiological features (Cho et al., 1997). Human infection primarily occurs through the consumption of raw or undercooked freshwater crabs or crayfish containing infective *P. westermani* metacercariae. Although the lungs are the primary site of infection, *P. westermani* may migrate to ectopic location.

Ectopic paragonimiasis has been well documented in organs such as the brain, spinal cord, heart, abdominal cavity, and subcutaneous tissues, reflecting the parasite's ability to disseminate beyond the lungs (Hong and Yong, 2020; Kim et al., 2022). These ectopic infections can lead to diagnostic confusion and clinical mismanagement, especially in non-endemic areas with limited physician awareness.

Globally, approximately 23 million people are infected with *Paragonimus* species, and an estimated 292 million people are at risk, primarily in East and Southeast Asia (Yoshida et al., 2019). In recent years, the increased consumption of imported freshwater crustaceans along with international travel and labor migration has led to sporadic cases of paragonimiasis in non-endemic regions. Several infections reported in the United States occurred in individuals with no history of travel to endemic areas and were linked to the consumption of raw, imported freshwater crabs served at local restaurants, including sushi establishments (Malvy et al., 2006; Boland et al., 2011).

In several countries, freshwater crustaceans are often widely obtained from Southeast Asia in marinated or frozen from (Singh et al., 2015). Although thorough cooking reliably inactivates paragonimus metacercariae, traditional preparation methods, such as soy sauce–marinated, salting, or alcohol pickling may fail to eliminate infectivity (Chai and Jung, 2022; Maekawa et al., 2023).

Several regional dishes have been implicated in paragonimiasis outbreaks, including “drunken crab” in China, “gejang” (soy sauce–marinated crab/crayfish) in Korea, “oboro-kiro” in Japan, “goong ten” in Thailand, and “kinuolao” in the Philippines (Chai and Jung, 2022). In Korea, *gejang*—soy sauce–marinated freshwater crabs or crayfish—has been recognized as a significant source of lung fluke infection (Cho et al., 1997). However, it is important to note that gejang made with marine crabs, which are commonly consumed and widely known as part of Korean cuisine, is unrelated to the transmission of *P. westermani*, as marine species are not involved in its life cycle.

Although freshwater crabs (e.g., Eriocheir japonica) are more commonly used in soy sauce–marinated dishes in Korea, freshwater crayfish (*Cambaroides similis*) are occasionally been used in local preparations. For practical reasons and because *C. similis* is a natural second intermediate host of *P. westermani* (Kim et al., 2009). Thus, for practical purposes, this species was selected as the experimental model in our study.

Although marination can inactivate metacercariae, its effectiveness depends on both salt concentration and duration. Higher salinity and prolonged exposure increase the likelihood of inactivation; however, *P. westermani* metacercariae have demonstrated the ability to survive for >1 week under certain conditions (Kim et al., 2017).

In South Korea, multiple cases of paragonimiasis have been reported following the consumption of freshwater crabs, involving both pulmonary and extrapulmonary presentations (Koh et al., 2012; Lee et al., 2012). According to Health Insurance Review and Assessment Service (2010−2023), an average of 115 cases of paragonimiasis were recorded annually.

Despite several reports on paragonimiasis, only a few studies have evaluated the infectivity of *P. westermani* metacercariae following traditional marination. This study aimed to assess the viability and infectivity of *P. westermani* metacercariae in freshwater crayfish (*Cambaroides similis*) marinated in soy sauce with varying salt concentrations and durations. We hypothesized that *P. westermani* metacercariae can survive high-salt marination and retain the infectivity posing a potential food safety risk. These findings are intended to inform food safety policies and raise awareness of the potential public health risk associated with traditional preparation methods.

## 2 Materials and methods

### 2.1 Collection of *P. Westermani* metacercariae from freshwater crayfish

**Fig. 1:**
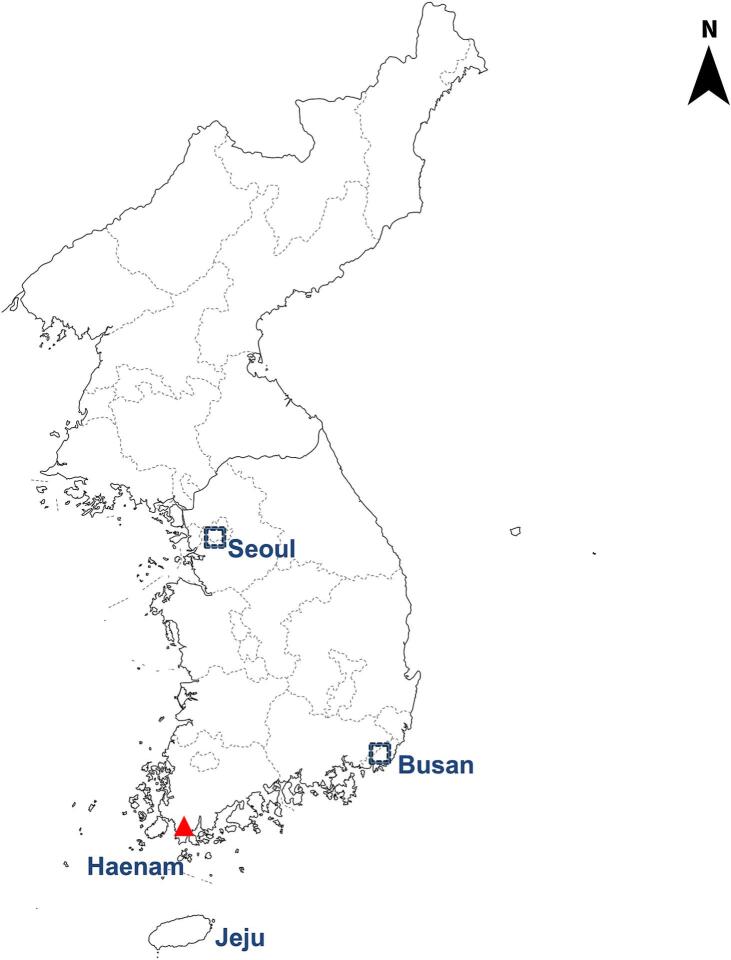
Collection site of freshwater crayfish (*Cambaroides similis*) in Haenam, Jeollanam-do, South Korea (34.5735°N, 126.5974°E). This area is known as an endemic region for *Paragonimus westermani.* The sampling site is indicated by a red triangle. (For interpretation of the references to colour in this figure legend, the reader is referred to the web version of this article.)

A total of 130 freshwater crayfish (*Cambaroides similis*), which are known intermediate hosts of *Paragonimus westermani*, were collected from Haenam, Jeollanam-do, South Korea and the geographical coordinates (34.5735°N, 126.5974°E) as shown in Fig. 1. The crayfish ranged from 5.4 to 7.6 cm in length (mean: 6.4 cm) and 2.8–5.2 g in weight (mean: 3.4 g). To confirm the presence of *P. westermani* metacercariae, 30 crayfish were randomly selected and examined. Each crayfish was crushed in a small bowl, suspended in 100 mL of tap water, and filtered through four layers of gauze. The residue was diluted in 100 mL of saline, re-filtered, and allowed to settle. The sediment was transferred to a Petri dish, and *P. westermani* metacercariae were identified under a stereomicroscope.

### 2.2 Soy sauce marination of infected crayfish

To assess the viability of *P. westermani* metacercariae following soy sauce marination, infected crayfish (*n* = 100) were incubated in commercial soy sauce (Sempio Foods Co., Seoul, Korea) adjusted to either 10 % or 20 % NaCl. Final salinity was confirmed using a salinity refractometer (MASTER-S28M, No. 2483, ATAGO, Japan). The crayfish were stored at 4 °C and sampled at multiple time points over a 1–60-day period. After each sampling time point, crayfish were randomly selected from each group and processed as described above. The isolated *P. westermani* metacercariae were stored in saline at 4 °C for subsequent infectivity testing. All experiments were performed in duplicate. Crayfish were evenly assigned to the 10 % and 20 % NaCl groups, which were tested in parallel throughout the marination period. Crayfish were examined on days 3, 5, 14, 30, 45, and 60 of marination to determine metacercarial viability at each time point.

### 2.3 Infectivity assessment and worm recovery

To assess infectivity, 100 *P. westermani* metacercariae from each group were orally inoculated into three beagle dogs (*n* = 3 per group). The worm recovery rate and worm recovery were evaluated after 12 weeks.

Recovered *P. westermani* metacercariae were morphologically categorized into three groups: viable, borderline (uncertain viability), and dead. For infectivity evaluation, three beagle dogs were orally inoculated with 100 *P. westermani* metacercariae from each group using a gastric gavage needle. Twelve weeks post-inoculation, the animals were euthanized, and adult worms were recovered from lung tissues. Worm recovery rates were calculated and expressed as mean ± standard deviation.

### 2.4 Statistical analysis

Statistical comparisons of *P. westermani* metacercariae viability across marination durations and salt concentrations were performed using the nonparametric Mann–Whitney *U* test. A *p*-value of <0.05 was considered statistically significant. All analyses were conducted using GraphPad Prism version 9.0 (GraphPad Software, San Diego, CA, USA).

## 3 Results

### 3.1 Detection of *P. Westermani* metacercariae in crayfish

Of the 130 crayfish used in this study, 30 were confirmed positive for *P. westermani* metacercariae. The number of metacercariae per crayfish ranged from 28 to 61, with a mean of 40.4 ± 6.3.

### 3.2 *P. Westermani* metacercariae viability after marination in soy sauce

The survival of *P. westermani* metacercariae in crayfish was affected by soy sauce salinity. Higher salinity and longer marination durations led to a more pronounced significantly reduction in *P. westermani* metacercariae viability (Fig. 1 and Table 1). After 14 days of margination, *P. westermani* survival rastes were 52.2 ± 4.1 % in 10 % NaCl soy sauce and 2.5 ± 1.8 % in 20 % NaCl soy sauce (*n* = 3 replicates of 8 crayfish each). Thus, even prolonged high-salt, cold-storage marination fails to fully inactivate *P. westermani* metacercariae.

**Table 1:** Viability of *Paragonimus westermani* metacercariae in crayfish marinated in soy sauce with 10 % or 20 % salinity.

Soy sauce salinity (%)	Days of crayfish marination	No. (%) of PwMc by the viability categories		
		Viable	Borderline	Dead
10	3	84.2 ± 3.7*	15.8 ± 3.2	0
	5	72.4 ± 4.2*	27.6 ± 5.1	0
	14	52.2 ± 4.1*	34.2 ± 6.3	13.6 ± 3.8
	30	40.6 ± 3.4*	33.4 ± 2.5	26 ± 2.3
	45	0	17.8 ± 2.2	82.2 ± 1.9
	60	0	0	100
20	3	67.3 ± 3.3*†	30 ± 2.5	2.7 ± 1.2
	5	28.8 ± 4.4*†	35.4 ± 4.1	35.8 ± 2.2
	14	2.5 ± 1.8*†	56.5 ± 2.2	41 ± 4.4
	30	0	0	100

Data represent viability (%) of P. westermani metacercariae from 8 crayfish per group.

p < 0.05 vs. day 0 within each salinity group (Mann–Whitney U test, *).

†*p* < 0.05 between salinity groups at the same time point (Mann–Whitney U test).

**Fig. 2:**
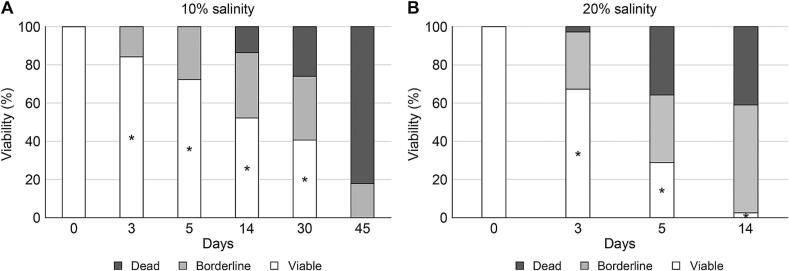
Viability of *Paragonimus westermani* metacercariae in crayfish marinated in soy sauce containing10 % (A) and 20 % (B) NaCl concentrations. Data represent means from 8 crayfish per time point. Asterisks (*) indicate statistically significant differences from day 0 within the same salinity group (Mann–Whitney *U* test, *P* < 0.05)*.

*P. westermani* metacercariae were classified into three categories based on viability, morphology and motility: viable (larval movement in cyst, uniform morphology, round/spherical shape with a thick inner cyst wall), borderline (no movement in cyst, thin inner cyst wall exhibiting soy sauce absorption), and dead (completely deteriorated layer, ruptured inner cyst wall) (Fig. 2).

In 10 % NaCl soy sauce, most *P. westermani* metacercariae were viable (84.2 ± 3.7 %) after 3 days, with borderline forms increasing over time (e.g., 34.2 ± 6.3 % on day 14). No viable metacercariae were detected after 45 days. In contrast, in 20 % NaCl soy sauce, viability decreased more rapidly- from 67.3 ± 3.3 % on day 3 to 2.5 ± 1.8 % on day 14-and no viable metacercariae were observed after 30 days. Borderline forms were predominant during the intermediate stages (e.g., 56.5 ± 2.2 % after day 14 in 20 % NaCl).

### 3.3 Worm recovery from dogs

**Fig. 3:**
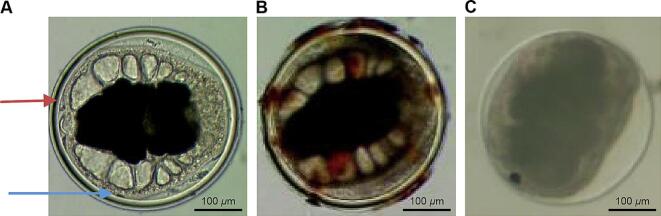
Morphological classification of *P. westermani* metacercariae (PwMc) after marination in 10 % NaCl soy sauce for 14 days. (A) Viable PwMc showing active movement and an intact thick cyst wall. The red arrow indicates the outer cyst wall; the blue arrow indicates the inner cyst wall. (B) Borderline PwMc with an intact cyst wall and partial deterioration, showing soy sauce absorption. (C) Dead PwMc with a collapsed or ruptured cyst wall. (For interpretation of the references to colour in this figure legend, the reader is referred to the web version of this article.)

**Fig. 4:**
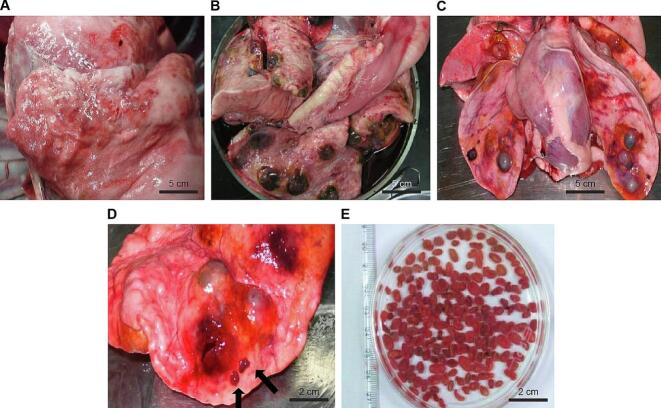
Infectivity of PwMc from each viability category following oral inoculation into dogs (*n* = 3). (A–C) Lung tissue of dogs infected with (A) dead, (B) viable, and (C) borderline PwMc after 12 weeks. (D) Mature *P. westermani* worms encapsulated in lung nodules; black arrows indicate emerging worms. (E) Adult worms collected from dogs infected with viable or borderline PwMc.

In pulmonary infections, *P. westermani* worms form cysts (nodules) of host origin, 2–3 cm in diameter, within the lung parenchyma. Dogs were orally infected with the three classification types of *P. westermani* metacercariae using a gavage needle and euthanized after 3 months. Worm recovery yielded mean values of 80.8 ± 5.1, 33.3 ± 4.4, and 0 mature worms in the viable, borderline, and dead groups repectively (*n* = 3 dogs per group). Notably, all dogs in the borderline group were infected, indicating that borderline *P. westermani* metacercariae despite morphological damage and soy sauce absorption-retained infectivity in all dogs, albeit with a significantly reduced worm burden compared to the viable group (Fig. 3 and Table 2). (See Fig. 4.)

**Table 2:** Worm recovery rates in dogs based on *Paragonimus westermani* metacercariae viability.

PwMc viability categories (%)	Number of PwMc	Number of worms
Viable	100	81 ± 4.5
Borderline	100	33 ± 8.5
Dead	100	0

Data are presented as means ± standard deviations from three dogs per group (n = 3).

## 4 Discussion

This study demonstrates that *P. westermani* metacercariae can survive for extended periods in soy sauce–marinated freshwater crayfish, even under high-salinity and refrigerated conditions. Importantly, *P. westermani* metacercariae extracted from these marinated samples retained infectivity when orally administered to dogs. These findings raise important public health concerns, particularly because cases of paragonimiasis continue to occur in non-endemic regions and are often associated with the consumption of imported or inadequately processed freshwater crustaceans (Diaz, 2013; Diaz et al., 2022). While general guidelines recommend parasite inactivation via freezing, heating or high-salt and alcohol-based marination (e.g., drunken crab), there are currently no established standards for soy sauce–based marination or for freshwater crabs and crayfish (FDA US, 2022).

The survival of *P. westermani* metacercariae appears to be influenced by multiple factors, including salinity, marination duration, and host species composition. In this study, complete inactivation was observed after 30 days in 10 % NaCl and 14 days in 20 % NaCl soy sauce. These concentrations were selected to reflect NaCl content of commercially available Korean soy sauces, which typically range from 10 % to 20 % NaCl. These concentrations also correspond to undiluted or lightly modified soy sauce commonly used in domestic preparation of raw-marinated crayfish, known as “ganjang-gejang.” Thus, our experimental conditions closely mirror local culinary practices. Interestingly, previous research reported shorter survival durations for *P. westermani* metacercariae in soy sauce. For example, Kim et al. (2017) observed complete inactivation of *P. westermani* metacercariae within 4 days in 10 % soy sauce. These discrepancies may be attributed to differences in experimental design-specifically, the use isolated metacercariae directly exposed to soy sauce in vitro in Kim et al. (2017), versus the use of naturally infected whole crayfish in our study, where the intact exoskeletons and internal tissue structures likely delayed marinated penetration, thereby prolonging survival.

Although increased salt concentration and prolonged marination reduced viability, infectivity was still observed when marinated crayfish were consumed before complete inactivation. Notably, borderline *P. westermani* metacercariae—characterized by reduced motility, cyst wall damage, and soy sauce absorption—retained infectivity. Following oral inoculation with 100 borderline *P. westermani* metacercariae, all three dogs became infected, and an average of 33 ± 8.5 adult worms were recovered per animal. This finding highlights the danger of assuming safety based solely on morphological appearance and underscore the limitations of visual assessment in determining parasite inactivation.

A limitation of our study was the assumption that all 30 crayfish used in the preliminary analysis were assumed to be infected with *P. westermani* metacercariae, which made it difficult to accurately estimate the initial metacercarial load in subsequent marination groups. Nonetheless, our data consistently demonstrate that *P. westermani* metacercariae, can persist for extended periods under high-salinity conditions and retain significant infectivity.

## 5 Conclusion

This study demonstrates that P. westermani metacercariae can remain viable and infectious in soy sauce–marinated freshwater crayfish, even under high-salt, refrigerated conditions. These findings highlight a clear route of foodborne transmission and reveal the inadequacy of traditional marination methods in inactivating infective stages. To prevent paragonimiasis, there is a critical need to revise and validate food safety guidelines that specifically address freshwater crustaceans prepared by non-thermal methods such as salting or soy sauce marination.These findings highlight a clear route of foodborne transmission and reveal the inadequacy of traditional marination methods in inactivating infective stages. To prevent paragonimiasis, there is a critical need to revise and validate food safety guidelines that specifically address freshwater crustaceans prepared by non-thermal methods such as salting or soy sauce marination.

## Availability of data and materials

All data generated or analyzed during this study are included in this published article.

## CRediT authorship contribution statement

**Eun-Min Kim:** Writing – review & editing, Writing – original draft, Visualization, Validation, Supervision, Software, Resources, Project administration, Methodology, Investigation, Funding acquisition, Formal analysis, Data curation, Conceptualization. **Yan Jin:** Writing – review & editing, Methodology. **Sung-Tae Hong:** Writing – review & editing, Supervision, Project administration, Funding acquisition, Conceptualization.

## Ethics statement

All animal procedures were approved by the Institutional Animal Care and Use Committee (IACUC) of Seoul National University, Seoul, Republic of Korea (approval no. SNU20080022), and were conducted in accordance with the National Institutes of Health (NIH) guidelines for the care and use of laboratory animals. No clinical signs of distress or abnormal behavior were observed in any animals during the 12-week observation period.

## Declaration of generative AI and AI-assisted technologies in the writing process

The authors declare that no generative AI or AI-assisted technologies were used in the writing of this manuscript.

## Funding

This study was supported by a research grant from the Korea Association of Health Promotion (2008–0001) and by the 10.13039/501100003725National Research Foundation of Korea (NRF), funded by the Korean government (RS-2023-00248109).

## Declaration of competing interest

The authors declare that they have no competing interests.
